# Diagnostic Performance of a Smart Device With Photoplethysmography Technology for Atrial Fibrillation Detection: Pilot Study (Pre-mAFA II Registry)

**DOI:** 10.2196/11437

**Published:** 2019-03-05

**Authors:** Yong-Yan Fan, Yan-Guang Li, Jian Li, Wen-Kun Cheng, Zhao-Liang Shan, Yu-Tang Wang, Yu-Tao Guo

**Affiliations:** 1 College of Medicine Nankai University Tianjin China; 2 Department of Cardiology Chinese People's Liberation Army General Hospital Beijing China; 3 Department of Geriatric Cardiology Chinese People's Liberation Army General Hospital Beijing China

**Keywords:** atrial fibrillation, photoplethysmography, detection, accuracy, mobile phone, smart band, algorithm

## Abstract

**Background:**

Atrial fibrillation (AF) is the most common sustained cardiac arrhythmia. The asymptomatic nature and paroxysmal frequency of AF lead to suboptimal early detection. A novel technology, photoplethysmography (PPG), has been developed for AF screening. However, there has been limited validation of mobile phone and smart band apps with PPG compared to 12-lead electrocardiograms (ECG).

**Objective:**

We investigated the feasibility and accuracy of a mobile phone and smart band for AF detection using pulse data measured by PPG.

**Methods:**

A total of 112 consecutive inpatients were recruited from the Chinese PLA General Hospital from March 15 to April 1, 2018. Participants were simultaneously tested with mobile phones (HUAWEI Mate 9, HUAWEI Honor 7X), smart bands (HUAWEI Band 2), and 12-lead ECG for 3 minutes.

**Results:**

In all, 108 patients (56 with normal sinus rhythm, 52 with persistent AF) were enrolled in the final analysis after excluding four patients with unclear cardiac rhythms. The corresponding sensitivity and specificity of the smart band PPG were 95.36% (95% CI 92.00%-97.40%) and 99.70% (95% CI 98.08%-99.98%), respectively. The positive predictive value of the smart band PPG was 99.63% (95% CI 97.61%-99.98%), the negative predictive value was 96.24% (95% CI 93.50%-97.90%), and the accuracy was 97.72% (95% CI 96.11%-98.70%). Moreover, the diagnostic sensitivity, specificity, positive predictive value, negative predictive value, and accuracy of mobile phones with PPG for AF detection were over 94%. There was no significant difference after further statistical analysis of the results from the different smart devices compared with the gold-standard ECG (*P*>.99).

**Conclusions:**

The algorithm based on mobile phones and smart bands with PPG demonstrated good performance in detecting AF and may represent a convenient tool for AF detection in at-risk individuals, allowing widespread screening of AF in the population.

**Trial Registration:**

Chinese Clinical Trial Registry ChiCTR-OOC-17014138; http://www.chictr.org.cn/showproj.aspx?proj=24191 (Archived by WebCite at http://www.webcitation/76WXknvE6)

## Introduction

Atrial fibrillation (AF) is the most common sustained cardiac arrhythmia encountered in clinical practice and is associated with increased risk of stroke, systemic embolism, heart failure, hospitalization, and death [1-3]. At least one-third of patients with AF are asymptomatic [4]. Due to the asymptomatic nature and paroxysmal frequency of this arrhythmia, early detection is challenging and often unsuccessful [5]. Strikingly, acute stroke or heart failure is often the first sign of AF [6,7]. Asymptomatic AF is also related with worse outcomes compared with symptomatic AF [8]. However, approximately 70% of AF-related strokes could be avoided with early detection and early management, such as initiation of oral anticoagulants [9]. The European Society of Cardiology now recommends screening of AF in the primary care of high-risk patients, including those aged 65 years and older, which is important for the prevention of stroke [10].

Although studies have shown that more frequent monitoring can improve AF detection [11-13], population-based screening remains suboptimal because of the inconvenience and high expense of current screening approaches, such as Holter monitoring with different time durations (24 hours and 7 days) [14,15]. In addition, the diagnostic value of the standard 12-lead electrocardiogram (ECG) is limited by the need for patients to obtain ECG diagnostic equipment, as well as the requirement that AF be present at the time of the ECG recording. As such, we need a more convenient heart rhythm screening or detection approach to identify AF and initiate early treatment.

Technical options to detect AF have significantly improved within the past decade. Different technologies such as blood pressure monitors [16], single-lead cardiac event monitors (Kardia Mobile case or card) [17], and mobile phone apps using photoplethysmographic signals have emerged for this purpose [18,19]. Taggar et al [20] analyzed 21 studies that investigated 39 interventions for detecting AF before March 16, 2015. Their meta-analysis found that the most accurate methods for detecting AF were blood pressure monitors, which had a sensitivity of 98% and a specificity of 92%, and non-12-lead ECG, which had a sensitivity of 91% and a specificity of 95%. Although blood pressure monitors and non-12-lead ECG both had relatively high diagnostic accuracies, the need for a blood pressure monitor and a clinical specialist to analyze the non-12-lead ECG pose a challenge for wide-scale implementation of AF screening. ECG hand-held devices usually provided a single-lead ECG for detecting AF, which including two different types: on-device diagnostic algorithm and transmitted data devices. According to the data from the sixth Atrial Fibrillation NETwork (AFNET) and the European Heart Rhythm Association (EHRA) Consensus Conference [21], the sensitivity of an on-device diagnostic algorithm was 94% to 98%, and the specificity was 76% to 97%. Transmitted data devices achieved a sensitivity and specificity of 94% to 96% and 90% to 95% for AF detection, respectively [21]. However, the diagnostic sensitivity of photoplethysmography (PPG) for AF detection was 97% to 100%, and the specificity was 92% to 94% [21]. The potential of PPG technology for AF detection has been confirmed in some small sample studies, but it is still necessary to verify the diagnostic accuracy of PPG technology for AF detection with a larger sample size study in the real world.

Available studies have indicated that PPG technology is a promising technology for AF screening. PPG is an optical method measuring changes in tissue blood volume through the skin capillary bed, which can be performed by using a mobile phone without any additional peripheral equipment [22,23]. The PPG waveform is generally acquired by the built-in camera of a mobile phone to measure pulsatile changes in light absorption reflected from a fingertip illuminated by the light-emitting diode (LED) flash [19]. Most smart bands currently on the market use PPG technology, and heart rate sensors on most smart bands work via PPG [24]. Detecting AF using easily accessible devices, such as mobile phones and smart bands, may represent a novel opportunity to passively and automatically detect asymptomatic AF that does not require additional hardware and is simple to operate. However, there has been limited validation of mobile phone and smart band PPG compared to 12-lead ECG, and the consistency and stability of PPG technology in AF screening with different smart devices still lack accurate data.

In this study, we tested the hypothesis that pulse waveform signals recorded using smart devices, including mobile phones and smart bands, can be analyzed by a realizable algorithm and can distinguish AF from normal sinus rhythm. This study also provides data on AF screening technology for the Chinese population.

## Methods

### Study Population

A total of 112 consecutive inpatients were recruited from the Chinese People's Liberation Army General Hospital (Beijing, China) from March 15 to April 1, 2018. Information regarding demographic characteristics, medical history, blood test results, and medications were recorded.

Patients aged 18 years and older were included in the study. Exclusion criteria included patients unable to use mobile phones and smart bands, with mental or memory problems, or with a pacemaker or implantable cardioverter defibrillator. Written informed consent was obtained and signed by each individual willing to take part in the study.

The mobile atrial fibrillation apps (mAFA) II registry is mobile health (mHealth) technology for improved screening, patient involvement, and optimizing integrated care of AF. Ours was a single-center pilot study of AF screening that was pre-mAFA II registry. The Medical Ethics Committee of the Chinese PLA General Hospital and the China Food and Drug Administration approved the study protocol (no: S2017-105-02). The study is registered in the Chinese Clinical Trial Registry, International Clinical Trials Registry Platform of the World Health Organization (ChiCTR-OOC-17014138).

### Signal Acquisition and Processing

Mobile phones (HUAWEI Mate 9, HUAWEI Honor 7X; Huawei Technologies Co, Ltd, Shenzhen, China) and smart bands (HUAWEI Band 2) were used for collecting pulse waveform signals. Pulse waveform recordings were performed by the participants under the supervision of trained study personnel. A dedicated data collection app, Heartbeats (Preventicus GmbH, Jena, Germany), was responsible for the pulse waveform signal acquisition and was installed in the HUAWEI mobile phones.

**Figure 1:**
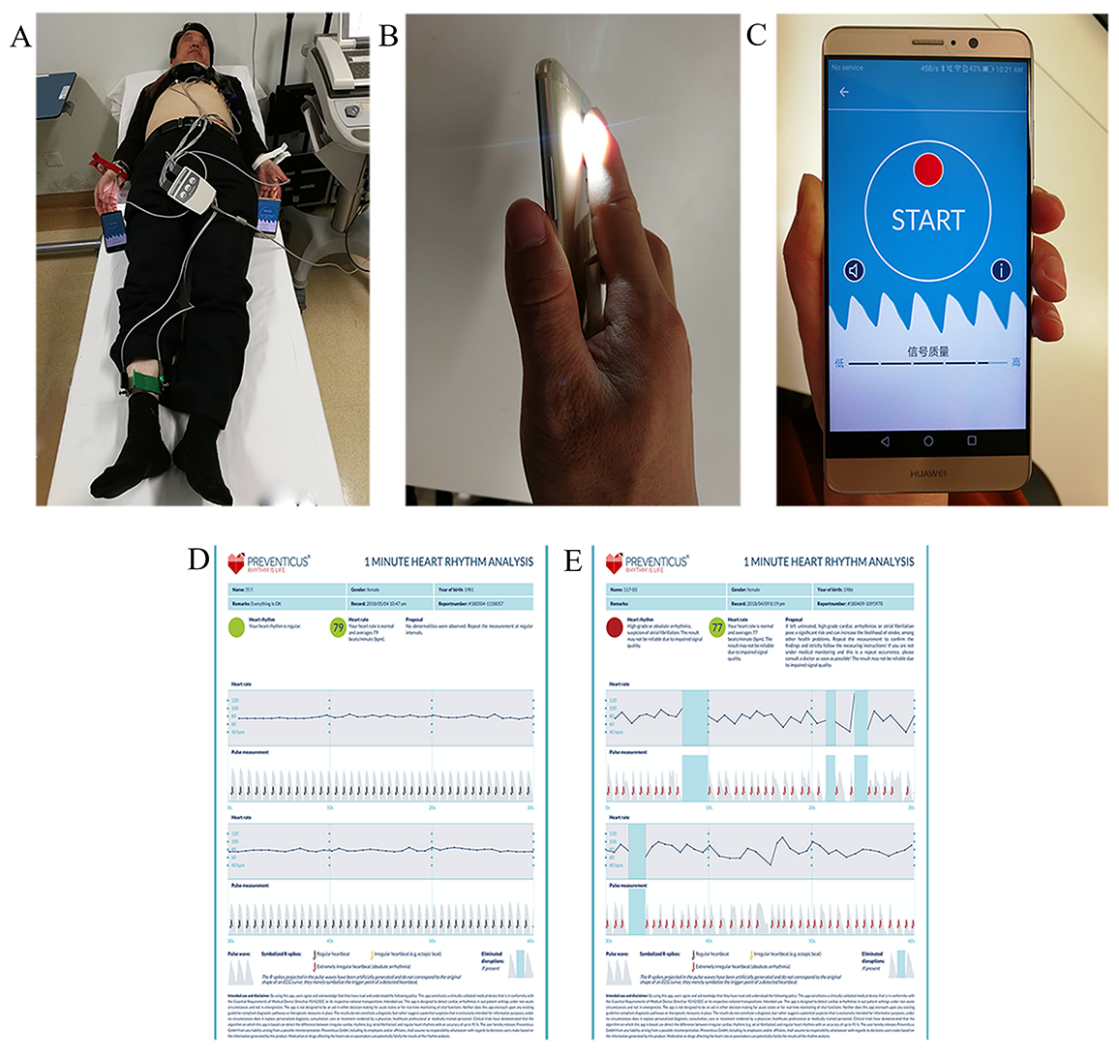
A prototype for atrial fibrillation detection using HUAWEI mobile phones and smart bands. A: A patient is simultaneously checked with HUAWEI mobile phones (Mate 9, Honor 7X), HUAWEI smart bands (Band 2), and 12-lead ECG. B: A fingertip is placed in contact with the built-in camera lens of a HUAWEI Mate 9 mobile phone and is illuminated by the adjacent LED flash. C: A screenshot of the pulse waveform data collection app (Heartbeats) running on a HUAWEI Mate 9 mobile phone. D: Representative pulse waveform recording from a patient with normal sinus rhythm. E: Representative pulse waveform recording from a patient with persistent atrial fibrillation.

Participants were simultaneously tested with mobile phones (HUAWEI Mate 9, HUAWEI Honor 7X), smart bands (HUAWEI Band 2), and 12-lead ECG for 3 minutes. Participants were advised to lie down in a supine position and breathe spontaneously. A HUAWEI Mate 9 (mobile phone 1) was positioned on the left-hand finger (either the index or middle finger) with the camera lens and LED light placed on the fingertip of the participant. Similarly, a HUAWEI Honor 7X (mobile phone 2) was positioned on the finger of the right hand. PPG measurements were performed by using the Heartbeats mobile phone app. The participant was asked to wear two smart bands, one each on the left and right hand. A 3-minute pulse waveform recording was obtained from each participant using the smart devices and a formal 12-lead ECG simultaneously. The PRO AF PPG was not running on the mobile devices in real time but remotely in the cloud. Then all 3-minute pulse waveform recordings using the smart devices were uploaded to the online cloud center and analyzed by a realizable algorithm (PRO AF PPG) provided by Preventicus (Preventicus GmbH, Jena, Germany). Figure 1 shows a prototype for AF detection using HUAWEI mobile phones and smart bands.

### Rhythm Diagnosis

Results from an ECG remain the gold standard for the measurement of heart rhythms, and they were confirmed by two independent cardiologists who were blinded to the baseline information of participants. The results of the algorithm were independently reviewed for each 12-lead ECG. For participants whose ECGs were initially affected by artifacts, trained study personnel instructed them to repeat the recordings to provide an optimal tracing for subsequent reading by the cardiologists. The Heartbeats app added a pulse waveform quality assessment step to reject recordings that were corrupted or too noisy and to prompt the user to retake a measurement.

**Figure 2:**
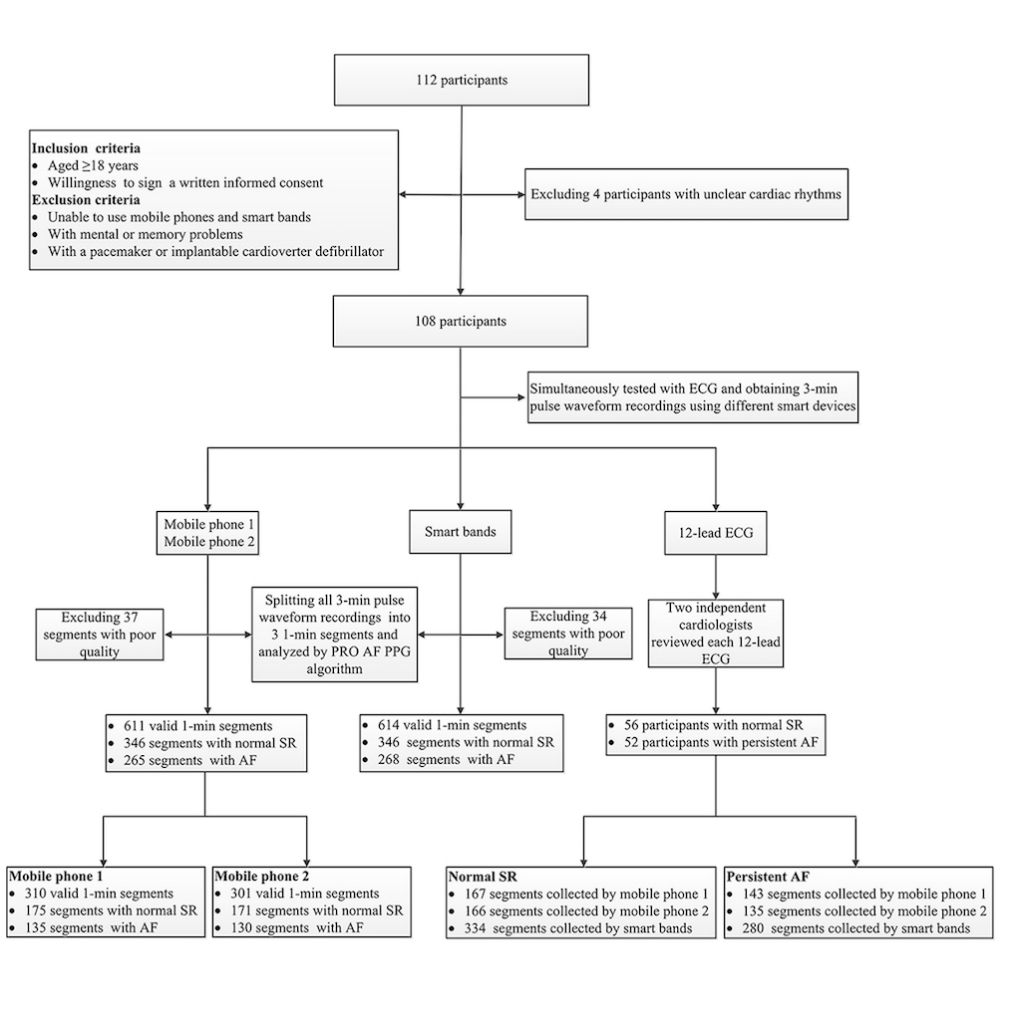
A flowchart of the study. AF: atrial fibrillation; ECG: electrocardiogram; SR: sinus rhythm.

After data collection, the novel heartbeat detection algorithm (PRO AF PPG), based on a combination of morphology and frequency analysis of the pulse waveform, was applied to perform beat-to-beat rhythm analysis and determine whether or not the participant suffered from AF. The diagnostic performance of the algorithm in detecting AF was then evaluated using the 12-lead ECG interpretation as the standard. Figure 2 shows a flowchart of the study.

### Statistical Analysis

Continuous variables were tested for normality by the Kolmogorov-Smirnov test. Data with a normal distribution are presented as means and standard deviations and were analyzed using the *t* test for two independent samples. Data with a nonnormal distribution are presented as medians and interquartile ranges (IQRs) and were analyzed using the Mann-Whitney *U* test. Data with discrete variables are presented as percentages and were analyzed using the Pearson chi-square test or Fisher exact test.

Sensitivity, specificity, positive predictive value, negative predictive value, and accuracy with 95% CI were used to measure the performance of our AF screening algorithm in the smart devices. The diagnostic performance of the algorithm in different devices was evaluated against reference ECG recordings, from which was calculated the number of true positives (TP), true negatives (TN), false positives (FP), and false negatives (FN). Sensitivity, specificity, positive predictive value, negative predictive value, and accuracy for AF diagnosis were calculated as simple proportions for the PRO AF PPG algorithm. The sensitivity was calculated as TP/(TP+FN) (true positives divided by all positives) and specificity as 1–FP/(TN+FP) (true negatives divided by all negatives). The corresponding positive predictive value was defined as TP/(TP+FP), and the negative predictive value as TN/(FN+TN). The corresponding accuracy was calculated as (TP+TN)/(TP+TN+FP+FN). Statistical evaluation was performed with SPSS 19.0 (SPSS Inc, Chicago, IL, USA). A value of *P*<.05 was considered statistically significant.

## Results

Among the 112 participants who fulfilled the inclusion criteria for the study, four participants were excluded because the ECG recordings showed unclear cardiac rhythms. As a result, 108 patients (56 with normal sinus rhythm, 52 with persistent AF) were enrolled in the final analysis. Table 1 summarizes the clinical characteristics of the study population. Participants with persistent AF were significantly older (*P*=.002), had a higher body mass index (*P*=.02), and had more prevalent heart failure (*P*=.006). Thromboembolic risk and bleeding risk were higher in participants with persistent AF compared to those with normal sinus rhythm based on CHA_2_ DS_2_-VASc score (median 3, IQR 2-5 vs median 2, IQR 1.3-3.75, *P*=.003) and HAS-BLED score (median 2, IQR 1-2 vs median 1, IQR 0-2, *P*=.005), respectively. The use of oral anticoagulants for preventing stroke was 77% (40/52) in participants with persistent AF and 18% (10/56) in participants with normal sinus rhythm (*P*<.001). The use of diuretics and digoxin were significantly higher in participants with persistent AF compared to those with normal sinus rhythm (*P*=.03 and *P*=.02, respectively), as well as the use of class III antiarrhythmic drugs (*P*<.001).

We split the 3-minute pulse waveform recordings of each participant obtained from mobile phones and smart bands into three 1-minute segments for further analysis with results from the 12-lead ECG. After splitting, there were 614 valid 1-minute segments of pulse waveform recordings in total obtained from smart bands, divided into 280 for AF and 334 for normal sinus rhythm based on ECG. Thirty-four 1-minute segments of signal recordings were deemed poor quality and were disregarded. The diagnostic performance of the PRO AF PPG AF screening algorithm in smart bands was evaluated against reference ECG recordings and demonstrated a sensitivity of 95.36% (95% CI 92.00%-97.40%) and a specificity of 99.70% (95% CI 98.08%-99.98%) for the detection of AF. The corresponding positive predictive value of the PRO AF PPG algorithm for AF screening was 99.63% (95% CI 97.61%-99.98%), the negative predictive value was 96.24% (95% CI 93.50%-97.90%), and the accuracy was 97.72% (95% CI 96.11%-98.70%).

For mobile phones, we obtained 611 valid 1-minute segments of pulse waveform recordings in total, of which 310 were obtained from mobile phone 1 and 301 from mobile phone 2, divided into 278 for AF and 333 for normal sinus rhythm based on standard ECG recordings. Thirty-seven 1-minute segments of signal recordings were omitted because of poor quality, of which 14 were recorded by mobile phone 1 and 23 by mobile phone 2. The diagnostic sensitivity and specificity of the PRO AF PPG algorithm for AF detection using mobile phone 1 and mobile phone 2 were 94.96% (95% CI 91.51%-97.11%) and 99.70% (95% CI 98.07%-99.98%), respectively. The positive predictive value was 99.62% (95% CI 97.59%-99.98%), the negative predictive value was 95.95% (95% CI 93.15%-97.68%), and the accuracy of the algorithm for AF detection was 97.55% (95% CI% 95.89%-98.57%). Moreover, the diagnostic sensitivity, specificity, positive predictive value, negative predictive value, and accuracy of mobile phone 1 alone or mobile phone 2 alone with PPG for AF detection were over 94%. Detailed diagnostic performance of the PPG technology for AF screening in different smart devices is summarized in Table 2. There was no significant difference in further statistical analysis of the results from different smart devices compared with ECG (*P*>.99).

Table 3 lists data from the literature on AF detection with different technologies. Compared with recent studies on AF detection, the PRO AF PPG algorithm showed good diagnostic performance from each smart device.

**Table 1:** Baseline characteristics of participants (N=108).

Characteristics		Sinus rhythm (n=56)	Persistent AF^a^ (n=52)	*P* value
**Demographics**				
	Age (years), mean (SD)	58 (14.78)	66.56 (13.17)	.002
	Female, n (%)	26 (46)	19 (37)	.30
	Body mass index (kg/m^2^), mean (SD)	24.44 (2.88)	25.98 (3.97)	.02
**Medical history**				
	Heart failure, n (%)	2 (4)	12 (23)	.006
	Hypertension, n (%)	29 (52)	35 (67)	.10
	Diabetes mellitus, n (%)	15 (27)	17 (33)	.50
	Previous stroke/SE^b^/TIA^c^, n (%)	4 (7)	9 (17)	.19
	Coronary artery disease, n (%)	25 (45)	19 (37)	.39
	Vascular disease, n (%)	31 (55)	37 (71)	.09
	COPD^d^, n (%)	1 (2)	3 (6)	.56
	Renal dysfunction, n (%)	2(4)	8 (15)	.07
	Hepatic dysfunction, n (%)	0	2 (4)	.23
	Sleep apnea, n (%)	2 (4)	6 (12)	.22
	Hyperthyroidism, n (%)	1 (2)	4 (8)	.32
	Current smoking, n (%)	16 (29)	17 (33)	.64
	Current drinking, n (%)	13 (21)	11 (23)	.80
	CHA_2_DS_2_-VASc^e^ score, median (IQR^f^)	2 (1-3.75)	3 (2-5)	.003
	HAS-BLED^g^ score, median (IQR)	1 (0-2)	2 (1-2)	.005
**Medications, n (%)**				
	Oral anticoagulant	10 (18)	40 (77)	<.001
	Antiplatelet drug	15 (27)	23 (44)	.06
	Calcium channel blockers	17 (30)	13 (25)	.54
	ACEI/ARB^h^	21 (38)	16 (31)	.46
	Diuretic	5 (9)	13 (25)	.03
	Digoxin	3 (5)	11 (21)	.02
**Antiarrhythmic drug, n (%)**				
	Class I	6 (11)	2 (4)	.32
	Beta blocker	27 (48)	34 (65)	.07
	Class III	3 (5)	20 (38)	<.001
	Class IV	3 (5)	3 (6)	>.99

^a^AF: atrial fibrillation.

^b^SE: systemic arterial embolism.

^c^TIA: transient ischemic attack.

^d^COPD: chronic obstructive pulmonary disease.

^e^CHA_2_DS_2_-VASc: congestive heart failure, hypertension, age ≥75 years, diabetes mellitus, stroke (doubled), vascular disease, age 65-74, female sex.

^f^IQR: interquartile range.

^g^HAS-BLED: hypertension, abnormal renal function, abnormal liver function, stroke, bleeding, labile INR, age >65 years, drugs or alcohol.

^h^ACEI/ARB: angiotensin-converting-enzyme inhibitor, angiotensin receptor blockers.

**Table 2:** Detailed diagnostic performance of the photoplethysmography technology for atrial fibrillation screening in different smart devices.

Index	Smart bands	Mobile phones	Mobile phone 1	Mobile phone 2
Sensitivity, % (95% CI)	95.36 (92.00-97.40)	94.96 (91.51-97.11)	94.41 (88.91-97.38)	95.56 (90.16-98.18)
Specificity, % (95% CI)	99.70 (98.08-99.98)	99.70 (98.07-99.98)	100 (97.20-100)	99.40 (96.18-99.97)
PPV^a^, % (95% CI)	99.63 (97.61-99.98)	99.62 (97.59-99.98)	100 (96.55-100)	99.23 (95.16-99.96)
NPV^b^, % (95% CI)	96.24 (93.50-97.90)	95.95 (93.15-97.68)	95.43 (90.88-97.86)	96.49 (92.17-98.57)
Accuracy, % (95% CI)	97.72 (96.11-98.70)	97.55 (95.89-98.57)	97.42 (94.78-98.80)	97.67 (95.05-98.97)

^a^PPV: positive predictive value.

^b^NPV: negative predictive value.

**Table 3:** Data from the literature on atrial fibrillation (AF) detection with different technologies.

Study, year, and population studied		AF detection protocol	Sensitivity, %	Specificity, %	PPV^a^, %	NPV^b^, %
**McManuset al, 2013 [25]**						
	76 patients before and after cardioversion	An iPhone 4S, an algorithm combining RMSSD^c^ and ShE^d^	96.2	97.5	—^e^	—
**Chan et al, 2016 [22]**						
	1013 patients	Cardiio Rhythm mobile phone app	92.9	97.7	53.1	99.8
	1013 patients	AliveCor automated algorithm	71.4	99.4	76.9	99.2
**Krivoshei et al, 2017 [19]**						
	80 consecutive patients	An iPhone 4S, an algorithm combining RMSSD and ShE	80	95	—	—
	80 consecutive patients	An iPhone 4S, an algorithm combining RMSSD and PPA^f^	95	95	—	—
	80 consecutive patients	An iPhone 4S, an algorithm combining ShE and PPA	50	95	—	—
**Rozen et al, 2018 [26]**						
	97 patients before and after electrical cardioversion	An iPhone, Cardiio Rhythm mobile app	93.1	90.9	92.2	92.0
**Bumgarner et al, 2018 [27]**						
	100 patients before and after cardioversion	Kardia Band from AliveCor paired with an Apple Smartwatch, AliveCor automated algorithm	93	84	—	—
**Tisonet al, 2018 [28]**						
	51 sedentary participants undergoing cardioversion	Smartwatch PPG^g^ coupled with a deep neural network	98	90.2	90.9	97.8
	1617 ambulatory participants	Smartwatch PPG coupled with a deep neural network	67.7	67.6	7.9	98.1

^a^NPV: negative predictive value.

^b^PPV: positive predictive value.

^c^RMSSD: root mean square of successive difference of RR intervals.

^d^ShE=Shannon entropy.

^e^Missing data.

^f^PPA: Poincaré plot analysis.

^g^PPG: photoplethysmography.

## Discussion

### Principal Findings

In this study, we demonstrated good diagnostic performance of the smart devices for AF detection using pulse waveform data measured by PPG. To the best of our knowledge, this is the first study on AF screening technology in a Chinese population. The main findings were (1) the PRO AF PPG algorithm demonstrated promising potential for accurate detection and discrimination of AF from normal sinus rhythm in a trial setting and may be applied to any mobile phone or smart band for AF screening, and (2) there was no significant difference in the results from different smart devices compared with ECG, and the tested models of mobile phone had no impact on the diagnostic performance of the algorithm.

In this study, HUAWEI mobile phones and smart bands showed higher consistency and stability of PPG technology in AF screening with PRO AF PPG algorithm and performed better with higher sensitivity and specificity. Prior to the development of the PRO AF PPG algorithm, several algorithms were validated for the detection of AF based on mobile phones and wearable devices (Table 3). Root mean square of successive difference of RR intervals (RMSSD), Shannon entropy (ShE), Poincaré plot analysis, and the AliveCor automated algorithm have been used to discriminate between AF and sinus rhythm by analyzing pulse waveform signals recorded using smart devices in several recent studies [19,22,25,27]. McManus et al [25] described an app using a camera and LED light of an iPhone 4S to record pulse waves obtained from the fingertips of patients. The signal recorded was processed through an algorithm combining RMSSD and ShE. They evaluated the algorithm in 76 patients before and after cardioversion, effectively using each patient as their own control, and reported a sensitivity of 96.2%, specificity of 97.5%, and accuracy of 96.8%. Krivoshei et al [19] applied the same published algorithm as McManus et al to detect AF with 80 consecutive patients. They demonstrated that the algorithm reliably discriminated between normal sinus rhythm and AF based on pulse wave signals from an iPhone 4S camera only and achieved a sensitivity and specificity of 80% and 95%, respectively. Rozen et al [26] conducted a study to assess the Cardiio Rhythm mobile phone app as a diagnostic tool in 97 patients before and after electrical cardioversion, and achieved a sensitivity of 93.1%, a specificity of 90.9%, a positive predictive value of 92.2%, and a negative predictive value of 92.0% for AF detection. Bumgarner et al [27] reported that the Kardia Band algorithm from AliveCor paired with an Apple Smartwatch accurately differentiated AF from sinus rhythm in 100 patients before and after cardioversion, and demonstrated 93% sensitivity and 84% specificity. Tison et al [28] demonstrated that smartwatch PPG coupled with a deep neural network can passively detect AF compared to standard 12-lead ECG among 51 sedentary participants undergoing cardioversion with a sensitivity of 98.0% and specificity of 90.2%, but with some loss of sensitivity (67.7%) and specificity (67.6%) in 1617 ambulatory participants.

Compared with other algorithms reported in previous studies, the PRO AF PPG algorithm performed better for AF screening in different smart devices with generally higher sensitivity, specificity, positive predictive value, negative predictive value, and accuracy. The majority of false positives originated from pulse waveforms that were corrupted by finger movement artifacts that may have affected the detection algorithm [22]. In fact, AF detection with single-lead ECG and PPG technology both should avoid the interference caused by movement to improve the accuracy. In this case, a 12-lead ECG was less affected by movement artifacts and may overall compare favorably in terms of inherent technical limitations compared to PPG. Although the diagnostic sensitivity of the PRO AF PPG algorithm was numerically lower compared to that from McManus et al [25] (95.56% vs 96.20%), this may be due to our different study design. In this study, we analyzed heart rhythm data from 108 consecutive inpatients, whereas McManus et al performed repeated measurements in the same individual patients before and after cardioversion. We consider our study design more reasonable and closer to the intended use of AF detection in a large-scale, high-risk population.

Although short-term pulse waveform recordings with mobile phones for AF screening are superior to single spot-checks in the clinic, they are vulnerable to misdiagnosis in many patients with paroxysmal AF. We attempted to partially address this limitation by using smart bands, which can be worn on the wrist for 24 to 48 hours or even longer to obtain long-term pulsatile PPG signals. Therefore, mobile devices, either mobile phones or smart bands, may provide at-risk patients with important tools for screening AF in a single shot or over a long duration, which may help facilitate the early detection and early management of asymptomatic AF before a devastating outcome such as ischemic stroke occurs.

### Strengths and Limitations

This is the first study on AF screening technology in China, and it demonstrates an easy AF screening approach using an algorithm based on smart devices with PPG and shows optimal diagnostic accuracy for heart rhythm readings. The advantage of AF screening with mobile phones is that it does not require additional hardware because optical video monitoring of the fingertip with a camera provides an accurate pulsatile time series related to variability in heart rate signals, making it more accessible and appealing to patients.

Some limitations of this study need to be addressed. First, we only focused on discriminating between AF and sinus rhythm in this study. However, the effect of sinus arrhythmia and other forms of ectopics, such as premature atrial beats, premature ventricular beats, and atrial flutter, on the performance of the algorithm should be evaluated, as these are common in the general population and might be similar in appearance to AF. In addition, the algorithm is unable to detect atrial flutter with a fixed atrioventricular conduction proportion that may also confer some risk of stroke and that frequently accompanies AF. Single-lead ECG may be used to detect AF and other arrhythmias. In our next study, new algorithms will be further developed to identify and differentiate sinus arrhythmia and various forms of ectopics. Second, although we reported the feasibility of using pulsatile PPG signals acquired from mobile phones and smart bands to detect AF in a group of participants preselected for their heart rhythm status, the ability to diagnose or screen AF with ambulatory outpatients has not been adequately investigated. Third, in this study, the data was collected in a supine position which could acquire stable pulse waveform signals and achieve reliable detection. However, the supine position is not common for home screening and creates inherent “not real world” rest conditions; therefore, it overestimates the results. Fourth, when we collected pulse waveform signals, we used the index or middle finger, not the pinky, which could have some effect on the results. However, it has been confirmed in several studies that using other fingers to collect pulse wave signals can also achieve good results [19,25,29]. Finally, although this was a pilot study, the sample size was relatively small, and more extensive clinical studies will need to be performed in the future.

### Conclusions

Atrial fibrillation can be detected and heart rhythms analyzed using the broadly accessible smart devices with PPG technology. It provides an accurate and easy method of discriminating AF form sinus rhythm and may be used to detect asymptomatic patients with AF. Although PPG technology can be used to detect AF with good performance, the final diagnosis of AF must still be based on ECG according to current guidelines. Further studies are needed to assess the efficacy of this approach in detecting, screening, and diagnosing AF early, which we are currently performing.

## Appendix

Multimedia Appendix 1 Baseline characteristics of participants.

Multimedia Appendix 2 Detailed diagnostic performance of the PPG technology for AF screening in different smart devices.

Multimedia Appendix 3 Data from the literature on atrial fibrillation detection with different technologies.

## Abbreviations

ACEI: angiotensin-converting-enzyme inhibitor
AF: atrial fibrillation
ARB: angiotensin receptor blockers
CHA_2_DS_2_-VASc: congestive heart failure, hypertension, age ≥75 years, diabetes mellitus, stroke (doubled), vascular disease, age 65-74, female sex
COPD: chronic obstructive pulmonary disease
ECG: electrocardiogram
FP: false positives
FN: false negatives
HAS-BLED: hypertension, abnormal renal function, abnormal liver function, stroke, bleeding, labile INR, age >65 years, drugs or alcohol
IQR: interquartile range
NPV: negative predictive value
PPA: Poincaré plot analysis
PPG: photoplethysmography
PPV: positive predictive value
RMSSD: root mean square of successive difference of RR intervals
SE: systemic arterial embolism
ShE: Shannon entropy
TIA: transient ischemic attack
TP: true positives
TN: true negatives
