# Supplementary material for: Diagnostic Performance of a Smart Device With Photoplethysmography Technology for Atrial Fibrillation Detection: Pilot Study (Pre-mAFA II Registry)
Source: JMIR Mhealth Uhealth. 2019 Mar 5;7(3):e11437. doi: 10.2196/11437 (PMC6423467; doi:10.2196/11437)
Supplement: Multimedia Appendix 1 [file mhealth_v7i3e11437_app1.pdf]

Table 1 Baseline characteristics of participants

Characteristics	Sinus rhythm (n=56)	Persistent AF (n=52)	*P* value
**Demographics**			
Age, mean ± SD	58 ± 14.78	66.56 ± 13.17	.002[a]
Female, n (%)	26 (46)	19 (37)	.298
Body Mass Index, kg/m^2, mean ± SD	24.44 ± 2.875	25.98 ± 3.974	.024[a]
**Medical history**			
Heart failure, n (%)	2 (4)	12 (23)	.006[a]
Hypertension, n (%)	29 (52)	35 (67)	.101
DM, n (%)	15 (27)	17 (33)	.502
Previous stroke/SE/ TIA, n (%)	4 (7)	9 (17)	.185
CAD, n (%)	25 (45)	19 (37)	.392
Vascular disease, n (%)	31 (55)	37 (71)	.089
COPD, n (%)	1 (2)	3 (6)	.558
Renal dysfunction, n (%)	2(4)	8 (15)	.074
Hepatic dysfunction, n (%)	0	2 (4)	.229
Sleep apnea, n (%)	2 (4)	6 (12)	.226
Hyperthyroidism, n (%)	1 (2)	4 (8)	.317
Current smoking, n (%)	16 (29)	17 (33)	.642
Current drinking, n (%)	13 (21)	11 (23)	.797
CHA_2DS_2-VASc score, median [IQR]	2[1-3.75]	3[2-5]	.003[a]
HAS-BLED score, median [IQR]	1[0-2]	2[1-2]	.005[a]
**Medications, n (%)**			
OAC	10 (18)	40 (77)	<.001[a]
Anti-platelet drug	15 (27)	23 (44)	.058
CCB	17 (30)	13 (25)	.535
ACEI/ARB	21 (38)	16 (31)	.461
Diuretic	5 (9)	13 (25)	.025[a]
Digoxin	3 (5)	11 (21)	.015[a]
Anti-arrhythmic drug			
Class I	6 (11)	2 (4)	.320

Beta-blocker	27 (48)	34 (65)	.072
Class III	3 (5)	20 (38)	<.001[a]
Class IV	3 (5)	3 (6)	1.00

[a] A *P* value less than .05 is considered as statistically significant.

AF=atrial fibrillation; SD=standard deviation; DM=diabetes mellitus; SE=systemic arterial embolism; TIA=transient ischemic attack; CAD=coronary artery disease; COPD=chronic obstructive pulmonary disease; CHA_2DS_2-VASc=congestive heart failure, hypertension, age≥75years, stroke (doubled), vascular disease, age 65-74, female sex; HAS-BLED=hypertension, abnormal renal function, abnormal liver function, stroke, bleeding, labile INR, age>65years, drugs or alcohol; OAC=oral anticoagulant; CCB=calcium channel blockers; ACEI/ARB=angiotensin-converting-enzyme inhibitor, angiotensin receptor blockers.
