# Supplementary material for: Diagnostic Performance of a Smart Device With Photoplethysmography Technology for Atrial Fibrillation Detection: Pilot Study (Pre-mAFA II Registry)
Source: JMIR Mhealth Uhealth. 2019 Mar 5;7(3):e11437. doi: 10.2196/11437 (PMC6423467; doi:10.2196/11437)
Supplement: Multimedia Appendix 2 [file mhealth_v7i3e11437_app2.pdf]

Table 2 Detailed diagnostic performance of the PPG technology for AF screening in different smart devices

Index	Smart bands	Smartphones	Smartphone 1	Smartphone 2
Sensitivity, % (95% CI)	95.36 (92.00-97.40)	94.96 (91.51-97.11)	94.41 (88.91-97.38)	95.56 (90.16-98.18)
Specificity, % (95% CI)	99.70 (98.08-99.98)	99.70 (98.07-99.98)	100 (97.20-100)	99.40 (96.18-99.97)
PPV, % (95% CI)	99.63 (97.61-99.98)	99.62 (97.59-99.98)	100 (96.55-100)	99.23 (95.16-99.96)
NPV, % (95% CI)	96.24 (93.50-97.90)	95.95 (93.15-97.68)	95.43 (90.88-97.86)	96.49 (92.17-98.57)
Accuracy, % (95% CI)	97.72 (96.11-98.70)	97.55 (95.89-98.57)	97.42 (94.78-98.80)	97.67 (95.05-98.97)

AF=atrial fibrillation; PPG=photoplethysmography; CI=confidence interval; PPV=positive predictive value; NPV=negative predictive value.
