# Supplementary material for: Diagnostic Performance of a Smart Device With Photoplethysmography Technology for Atrial Fibrillation Detection: Pilot Study (Pre-mAFA II Registry)
Source: JMIR Mhealth Uhealth. 2019 Mar 5;7(3):e11437. doi: 10.2196/11437 (PMC6423467; doi:10.2196/11437)
Supplement: Multimedia Appendix 3 [file mhealth_v7i3e11437_app3.pdf]

Table 3 Data from the literature on atrial fibrillation detection with different technologies.

Study and year	Population studied	AF detection protocol	Sensitivity (%)	Specificity (%)	PPV (%)	NPV (%)
McManus et al, 2013	76 patients before and after cardioversion	An iPhone 4S, an algorithm combining RMSSD and ShE	96.2	97.5	-	-
Chan et al, 2016	1013 patients	Cardiio Rhythm smartphone application	92.9	97.7	53.1	99.8
	1013 patients	AliveCor automated algorithm	71.4	99.4	76.9	99.2
Krivoshei et al, 2017	80 consecutive patients	An iPhone 4S, an algorithm combining RMSSD and ShE	80	95	-	-
	80 consecutive patients	An iPhone 4S, an algorithm combining RMSSD and PPA	95	95	-	-
	80 consecutive patients	An iPhone 4S, an algorithm combining ShE and PPA	50	95	-	-
Rozen et al, 2018	97 patients before and after electrical cardioversion	An iPhone, Cardiio Rhythm Mobile Application	93.1	90.9	92.2	92

Bumgarner et al, 2018	100 patients before and after cardioversion	Kardia Band from AliveCor paired with an apple smartwatch, AliveCor automated algorithm	93	84	-	-
Tison et al, 2018	51 sedentary participants undergoing cardioversion	smartwatch PPG coupled with a deep neural network	98	90.2	90.9	97.8
	1617 ambulatory participants	smartwatch PPG coupled with a deep neural network	67.7	67.6	7.9	98.1

AF=atrial fibrillation; PPV=positive predictive value; NPV=negative predictive value; RMSSD=root mean square of successive difference of RR intervals; ShE=Shannon entropy; PPA=Poincaré plot analysis; PPG=photoplethysmography.
